# Time-series clustering of gene expression in irradiated and bystander fibroblasts: an application of FBPA clustering

**DOI:** 10.1186/1471-2164-12-2

**Published:** 2011-01-04

**Authors:** Shanaz A Ghandhi, Anshu Sinha, Marianthi Markatou, Sally A Amundson

**Affiliations:** 1 Center for Radiological Research, Columbia University, VC11-215, 630 West 168th Street, New York, NY, 10032, USA; 2 Department of Biomedical Informatics, Columbia University, New York, NY 10032, USA; 3 Department of Biostatistics, Columbia University, New York, NY 10032, USA; 4 Department of Statistical Sciences, Cornell University, 301 Malott Hall, Ithaca, NY 14853-3801, USA

## Abstract

**Background:**

The radiation bystander effect is an important component of the overall biological response of tissues and organisms to ionizing radiation, but the signaling mechanisms between irradiated and non-irradiated bystander cells are not fully understood. In this study, we measured a time-series of gene expression after α-particle irradiation and applied the Feature Based Partitioning around medoids Algorithm (FBPA), a new clustering method suitable for sparse time series, to identify signaling modules that act in concert in the response to direct irradiation and bystander signaling. We compared our results with those of an alternate clustering method, Short Time series Expression Miner (STEM).

**Results:**

While computational evaluations of both clustering results were similar, FBPA provided more biological insight. After irradiation, gene clusters were enriched for signal transduction, cell cycle/cell death and inflammation/immunity processes; but only FBPA separated clusters by function. In bystanders, gene clusters were enriched for cell communication/motility, signal transduction and inflammation processes; but biological functions did not separate as clearly with either clustering method as they did in irradiated samples. Network analysis confirmed p53 and NF-κB transcription factor-regulated gene clusters in irradiated and bystander cells and suggested novel regulators, such as KDM5B/JARID1B (lysine (K)-specific demethylase 5B) and HDACs (histone deacetylases), which could epigenetically coordinate gene expression after irradiation.

**Conclusions:**

In this study, we have shown that a new time series clustering method, FBPA, can provide new leads to the mechanisms regulating the dynamic cellular response to radiation. The findings implicate epigenetic control of gene expression in addition to transcription factor networks.

## Background

Radon is the largest component of natural background radiation in the United States, and exposure is a risk factor for lung cancer. Comparison of epidemiological studies of uranium miners exposed to high levels of radon with studies of domestic exposures suggest that lower doses may be proportionately more dangerous than extrapolation from high doses would predict. This has resulted in the addition of a correction factor to domestic radon risk estimates, although the biological basis for this correction is not well understood [1]. As few cells sustain the direct traversal of a radon alpha particle at domestic exposure levels, non-targeted effects such as bystander response may increase the number of cells at risk [2] through mechanisms such as tumor promotion [3] or induction of genomic instability [4].

The radiation bystander effect is the response of cells in contact with or in the vicinity of irradiated cells. Many endpoints have been measured in bystander cells, including sister chromatid exchanges, micronuclei, apoptosis, terminal differentiation, mutation and gene expression changes [5-9]. Some of these outcomes might be considered protective, while others could increase tissue risk and a better understanding of the regulation of bystander responses is needed. The mechanisms of the bystander response are known to involve both direct cell-to-cell communication and indirect release of factors into extra-cellular space. A variety of signaling molecules, including cytokines, reactive oxygen species, nitric oxide, prostaglandins and MAPK (mitogen-activated protein kinases) have been shown to be implicated in the bystander response, but the signal transduction pathways that regulate bystander responses are still not clear [10].

Overall, radiation effects at the tissue and organism levels are complicated to understand because they occur at different levels of biological organization, from chromosomal damage to metabolic pathways [11]. After irradiation, signaling pathways rapidly modulate gene expression, which leads to additional signaling in the cell population both as a response to the initial damage and to maintain tissue homeostasis while the damage is being repaired [12]. Also, bystander effects can result in long-term genomic instability, which suggests that bystanders may continue to respond to signals for many generations after the initial irradiation event [13,14]. The radiation bystander effect, therefore, involves a complex cellular response across physical space and time. In the clinical context, the bystander effect has been linked with abscopal effects [15] and could potentially be exploited to enhance tumor-killing effects and to protect normal tissue from radiation exposure [12,16]. After irradiation, when the processes of tissue homeostasis are severely impaired, carcinogenesis has been demonstrated in unexposed bystander tissue [17] underlining the importance of understanding the mechanisms involved. Bystander responses are, therefore, especially relevant to cancer risk assessment in low-dose/low dose-rate radiation exposure situations such as domestic radon exposure or extended space travel, and also in partial body exposures such as from medical radiation.

It is important to understand not only the physiological and DNA damage effects of radiation on cells but also the global inflammatory and stress responses of cells and tissues. For instance, irradiated fibroblasts are known to promote tumor formation in neighboring epithelial cells by altering the tumor microenvironment [18]. With this in mind, we studied gene expression over time in normal human lung fibroblasts, at the mRNA level, to provide insight into the mechanisms and timing of signaling in irradiated and bystander cells. We have previously studied the gene expression response of bystander fibroblasts to 0.5 Gy α-particle irradiation, 4 hours after exposure [9]. To better understand both early and sustained signaling associated with responding genes, we have now extended the study, measuring global gene expression at 0.5 hour, 1 hour, 2 hours, 4 hours, 6 hours, and 24 hours after irradiation. We studied the direct radiation and bystander gene expression responses separately to compare trends because, although much is known about the effects of radiation on gene expression in cells [19], the full effect of radiation encompasses cells that are hit and those that are not. Also, over time the response in tissues comes from the convergence of signaling and responding genes from both types of cells. In the previous study of the 4 hour response, we identified 238 genes that were significantly changed 4 hours after exposure in irradiated and/or bystander cells [9]. In the current study, we focused our analysis on the response of these genes over time, and applied a novel time course clustering technique to identify genes with potential regulatory similarities.

The choice of methodology is a crucial issue in the use of clustering methods to examine structure in a given data set. It is important to choose and/or devise a methodology appropriate for the given data. Time series data are often analyzed using standard clustering algorithms such as hierarchical clustering, k-means and self-organizing maps [20-22]. Although these algorithms have yielded biological insights, the fundamental problem is that these methods typically treat measurements taken at different time points as independent, ignoring the sequential nature of time series data [23]. Furthermore, most methods that have been developed specifically for time course data [24-26] are designed for longer time series. In contrast, most microarray-based studies encompass relatively few time points. In this study, six time points and four biological replicates were measured, yielding sparsity in both the number of time points and the number of replicates. This characteristic rules out any modeling based on classical time-series methods, because there are an insufficient number of observations to allow accurate estimation of the parameters associated with the models. While short time series datasets such as presented here are becoming more common, there are still few choices for clustering that are tailored towards this type of data.

Here, we examine the data using two non-parametric clustering algorithms. The first is the Short Time series Expression Miner (STEM) algorithm and software developed by Ernst et al., where all genes are clustered into one of a set of pre-defined patterns based on transformation of gene profiles into "units of change" [23]. Then, clusters are assigned significance levels using a permutation test based method. Second, we apply a clustering method proposed in [27] that uses the Partitioning Around Medoids (PAM) [28] algorithm, which we have called the Feature Based PAM Algorithm (FBPA). It employs an innovative set of features of gene expression over time, such that, the unit of analysis changes from gene expression at given time points to profile curves over the entire time horizon. Unlike alternative approaches, it does not pre-specify patterns of expression and does not cluster point values using a distance measure or a model. The algorithm clusters biologically relevant features or curve summarization measures, extracted from each short time series, and then feeds these features into the PAM algorithm. PAM is very similar to the k-means algorithm, chosen here because it uses median data points to determine cluster centroids instead of the mean, making it more robust to outliers. This approach is designed to be both statistically powerful and biologically valid.

The idea of feature selection was first used in the context of clustering large time series data for dimension reduction, where the term dimension refers to the number of time points that describe the series. In these cases, a few well chosen statistics describing the dynamics of the series such as serial correlation, skewness, and kurtosis were used to summarize the data [29]. We also used feature selection, but in the sparse-data context, as a dimension augmentation technique to effectively and appropriately describe the curve and provide the most complete description of the time series possible. The clustering features we proposed here were based on the structural characteristics of the time course data and reflect a clear link with subject-matter considerations and the questions under study. The features we used were: the vector of slopes between adjacent time points, maximum and minimum expression, time of maximum and minimum expression, and the steepest positive and negative slope. In a sense, they capture the "global picture" of an admittedly short time horizon of expression and provide sufficient summarization of the dynamic structure of the curves. An obvious advantage of this method is that it can handle time series of various lengths with measurements taken at different time points as well as data with missing values. Although the fundamental idea on which this method is based, effective summarization of time course data, is transferable to a variety of application domains, the best features describing the time series are context-dependent and may differ depending on the application domain.

FBPA sufficiently describes the time course by performing dimension augmentation using biologically relevant features, thus avoiding interpolation/extrapolation; as such, the unit of the analysis is the time course itself, and not the expression measurements obtained at each time point. Because FBPA clusters all genes, it preserves information and renders unnecessary the notion of cluster significance. The use of biologically relevant features, together with the sufficient description of the time course, tends to produce clusters with focused biology.

This study addressed the question: can we extract information about regulation of genes in irradiated and bystander cells from closely coordinated temporal gene expression profiles? To do this we evaluated STEM and FBPA in both treatment conditions and showed our assessment of the results of both methodologies using computational measures as well as biological enrichment. To measure cluster tightness, we used homogeneity, and to measure cluster separation and structure we used the average silhouette, both are described in detail in the Methods section. To compare agreements of the various clustering methods, we used the Rand Index. We also curated a manual clustering using a subset of the data to compare clustering methods. We then assessed the biological implications of temporal clustering in both treatments and by both clustering methods, using gene ontology and pathway tools. Gene ontology analyses using the PANTHER tool showed that FBPA tended to cluster genes with related functions together and separated different biological processes into distinct clusters. This suggested that the features selected to describe the gene expression curves for FBPA analysis were more relevant to the underlying biological signaling than the parameters used in STEM. Network analysis using the Ingenuity Pathway Analysis (IPA) tool was also applied to the clusters enriched in related biological processes to identify potential hubs regulating specific aspects of the radiation and bystander responses. The overall picture of biological networks in irradiated versus bystander cells analyzed by FBPA clustering showed that temporal curves of gene expression after irradiation can be clearly differentiated into focused biological clusters. In comparison, bystander gene expression suggested that there is a general stress and inflammatory response in bystanders that can overshadow specific signaling networks. Some important and novel regulatory processes were suggested by the FBPA clustering approach, however, and we predicted the possible epigenetic regulation of the metallothionein gene family after irradiation and in neighboring bystanders as a novel finding in our study.

## Results and Discussion

### Microarray gene expression profiles and validation by qRT-PCR

**Figure 1:**
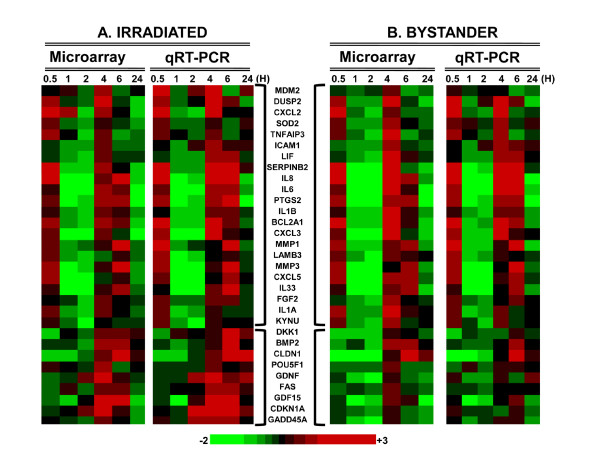
**Validation of gene expression measurements**. Heat-maps of log_2 _transformed expression ratios in (A.) irradiated and (B.) bystander cells to time matched sham-irradiated controls across the time points in hours (H) using both microarray and quantitative real time PCR methods. On the scale bar, red indicates up-regulation and green, down-regulation of mRNA compared to controls. Two major clusters of genes are evident: the biphasic response group with two distinct up-regulation peaks synchronously activated in both conditions, and 4-hour peak response genes in irradiated cells with a muted response in bystanders. Data are medians across four independent biological replicates.

We used Agilent whole human genome microarrays to measure relative gene expression in IMR-90 human fibroblast cells exposed to 0.5 Gy α-particles and in their bystanders at 0.5, 1, 2, 4, 6 and 24 hours post exposure. The data set was comprised of three treatment conditions (mock-irradiated controls, irradiated and bystander cells) at six time points, with four biological replicates of each condition. The data (GEO accession number GSE21059) were background corrected but not normalized in order to preserve dependence across time points. We have previously reported on the analysis of data at the 4-hour time point [9], and took the 238 genes responding significantly in that study as the focus for the present analysis. Forty of these genes were selected on the basis of the lowest FDR for differential expression in the microarray analysis, and were analyzed by quantitative real-time reverse-transcription PCR (qRT-PCR) to validate microarray measurements. The heat-maps in Figure 1 depict the qRT-PCR data as hierarchically-clustered logarithmically-transformed median gene expression ratios after irradiation and bystander treatment. Figure 1 also shows close concordance between ratios obtained by the microarray and qRT-PCR platforms. Overall, we found that qRT-PCR methods can give higher expression ratios as compared with microarray measurements, as reported previously [30]. We also confirmed previously observed gene expression patterns [9] in irradiated and bystander treated samples. One such pattern was the biphasic response of a large group of inflammatory/cytokine genes, including interleukin genes (*IL1β, IL6, IL8 *and *IL33*) and chemokine ligand genes (*CXCL3, CXCL5 *and *CXCL2*). The other pattern, a response of cell cycle and DNA damage genes (*CDKN1A, GADD45A *and *GDF15*) reaching maximum at 4-6 hours after treatment, was more pronounced in irradiated samples (Figure 1). Among the subset of genes analyzed here it was evident that there was more than one group of coordinately regulated genes, leading to our interest in developing an approach to group temporal profiles of gene expression in order to provide insight into regulatory nodes that may coordinately control gene expression.

### Manually curating clusters for comparison/validation of clustering methods

To evaluate the quality of clustering between methods, we manually curated clusters. Of 80 possible microarray profiles confirmed by qRT-PCR, 67 were selected, on the basis of pattern and known pathway information, as distinct and were grouped into seven clusters: no early peak; no change; two peaks and two dips; two peaks and two dips with a shallow second dip; two peaks and one dip with a low magnitude first peak; two peaks and one dip with a high magnitude first peak; and down at 4 hours. The graphs in Additional File 1 depict the results of manually curated clustering.

### Clustering gene expression after direct irradiation

**Figure 2:**
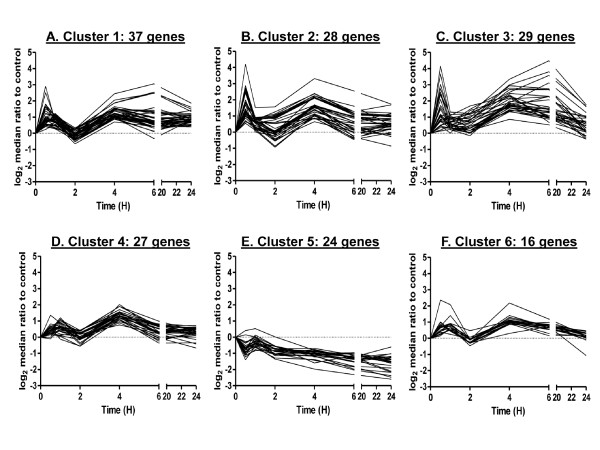
**STEM clustering on gene expression after direct exposure to 0.5 Gy α-particles**. Six significant clusters resulting from c = 2 and m = 50 are displayed as time course plots of median log_2 _gene expression ratios in irradiated vs. non-irradiated controls. The number of genes/curves in each cluster is shown. Data are medians across four independent biological replicates. Time is shown in hours (H).

**Figure 3:**
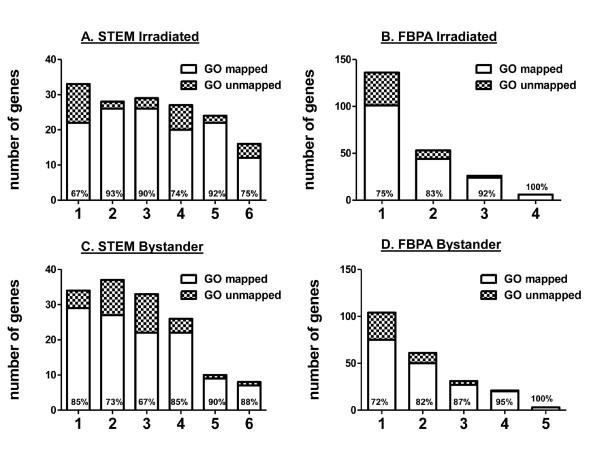
**Gene ontology mapping using the PANTHER database**. Genes from each of the STEM and FBPA clusters were mapped to annotations in the PANTHER database. Stacked bar-charts display the number of un-mapped (hatched bars) and mapped genes (white bars) in (A.) STEM clusters of gene response in irradiated cells; (B.) FBPA clusters of gene response in irradiated cells; (C.) STEM clusters of gene response in bystander cells and (D.) FBPA clusters of gene response in bystander cells. The percentage of genes annotated by PANTHER is indicated in the open bars (mapped genes). The cluster number is indicated on the x-axis. The total number of genes in each cluster represents the number of identifiers remaining after duplicate identifiers were removed. This adjustment is made because gene ontology methods do not recognize splice variants of genes and encoded variant proteins. In some cases this results in fewer genes than the input list from annotated microarray data.

We next used the STEM platform to cluster temporal profiles of gene expression in cells exposed to irradiation. After examining several combinations of input parameters, we found results to be relatively consistent across input parameters and selected results from c = 3 and m = 50 for further analysis of the irradiated data, where c indicates units of change and m, the number of candidate profiles. This run significantly clustered 174 out of the 238 cases (73.1% of 238 genes; Additional File 2). Figure 2 shows gene expression profiles for the six clusters found to be significant out of 50 possible clusters. The Rand Index to the manually curated clustering was 0.64, indicating good similarity (Table 1). The cardinality of each cluster was relatively uniform, ranging from 18 genes in cluster 6 to 37 genes in Cluster 1 (Figure 3A). Visual examination of the clusters suggested that biphasic responding genes were distributed across the first four clusters and that Cluster 3 also included genes that showed the more gradual increase, which peaked at 4 to 6 hours (Figure 2C). STEM also clustered down-regulated genes into a separate cluster, Cluster 5.

**Table 1:** Between-methods clustering agreement measured by RAND index in irradiated treatment and bystander response.

IRRADIATED					
	**Manually curated**	**Microarray FBPA**	**qRT-PCR FBPA**	**Microarray STEM**	**qRT-PCR STEM**
**Manually curated**	1	0.622	0.607	0.640	0.486
**Microarray FBPA**		1	0.681	0.596	0.533
**qRT-PCR FBPA**			1	0.706	0.615
**Microarray STEM**				1	0.644
**qRT-PCR STEM**					1

**BYSTANDER**					

	**Manually curated**	**Microarray FBPA**	**qRT-PCR FBPA**	**Microarray STEM**	**qRT-PCR STEM**
**Manually curated**	1	0.745	0.661	0.742	0.483
**Microarray FBPA**		1	0.742	0.671	0.561
**qRT-PCR FBPA**			1	0.643	0.615
**Microarray STEM**				1	0.490
**qRT-PCR STEM**					1

Gene expression of the 238 genes differentially expressed after irradiation was also clustered using FBPA on gene expression data features. To determine the optimal number of clusters, we used the gap statistic [31]. Where k is the number of clusters, we examined k = 4, 8, and 11, which all showed near zero inequalities. The average homogeneity was 3.026 and the average silhouette was 0.558 for k = 4 (Table 2). For k = 8, the average homogeneity was 2.098 and average silhouette was 0.434 (data not shown). With k = 11, average homogeneity was 1.764 and average silhouette was 0.371 (data not shown). Because good homogeneity and strong separation and structure were found with k = 4, we chose this clustering (Additional File 3). We note here that we tended towards parsimonious clustering as much as possible to avoid over-fitting the data and to group information that may be biologically relevant. The Rand Index to the manually curated standard was 0.623 (Table 1) also indicating good similarity, equivalent to that of STEM clustering on the microarray data after irradiation.

**Table 2:** FBPA cluster statistics on gene expression after direct irradiation

Cluster	Cardinality	Homogeneity	Average Silhouette
1	145	2.641	0.505
2	56	3.934	0.763
3	31	3.253	0.388
4	6	2.659	0.809
Total	238	3.026	0.558

Cluster cardinality is the number of genes in each cluster, homogeneity and average silhouette measurements are described in the Methods

**Figure 4:**
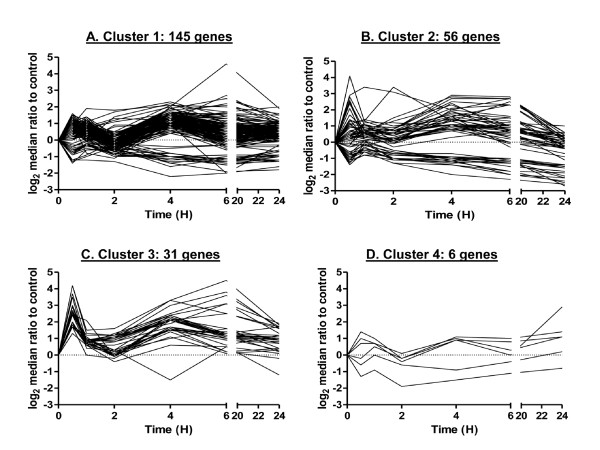
**FBPA clustering on gene expression after direct exposure to 0.5 Gy α-particles**. The four clusters resulting from k = 4, where k is cluster number, are displayed as time course plots of median log_2 _gene expression ratios in irradiated vs. non-irradiated controls. The number of genes/curves in each cluster is indicated. Data are medians across four independent biological replicates. Time is shown in hours (H).

Figure 4 shows the gene expression profiles clustered using FBPA. The within-method metrics (Table 2) gave interesting information. Because the method chose a small number of clusters, homogeneity was not strong, with the average homogeneity being close to 3. However, all but Cluster 3 showed good separation. The average silhouette over all clusters was 0.558 indicating that strong structure was found. We also noted that genes were not uniformly distributed across all clusters. In irradiated samples, 61% of the total number of genes clustered belonged to Cluster 1, 24% to Cluster 2, 13% to Cluster 3 and 2% belonged to Cluster 4 (Figure 4). Given that these genes were pre-selected on the basis of response at 4 hours, the clustering of a large proportion of genes together in one cluster in directly irradiated cells is not unexpected, because cells respond robustly to irradiation and transcripts of many of the genes included in this study could be affected in concert. It is also well known that many of the key radiation-response genes reach maximum expression at around 4 hours after treatment [32], as captured in Cluster 1 (Figure 4A and Additional File 3).

FBPA clusters showed more noise than STEM clusters, because all 238 genes were clustered. However, there appeared to be a general mapping between STEM and FBPA clusters. STEM Clusters 1, 4, and 6 mapped well to FBPA Cluster 1 (78.4%, 75%, 66.7% match respectively). STEM Cluster 2 mapped to FBPA Clusters 1 and 3 (41.7% and 50% match respectively). STEM Cluster 3 mapped partially to FBPA Clusters 1 and 2 (45.1% match and 41.9% match respectively). FBPA Cluster 4, however, did not match any of the STEM clusters. Also, genes showing down regulation, represented in STEM Cluster 5, were included in FBPA Clusters 1 and 2 (29.1% and 70.9%, respectively). Because the features selected for clustering (see Methods) did not emphasize magnitude of expression but rather rates of change, the down-regulated genes did not cluster separately in FBPA. Interestingly, all significant STEM clusters showed some degree of mapping to the largest FBPA cluster, Cluster 1.

### Clustering gene expression in the bystander response

**Figure 5:**
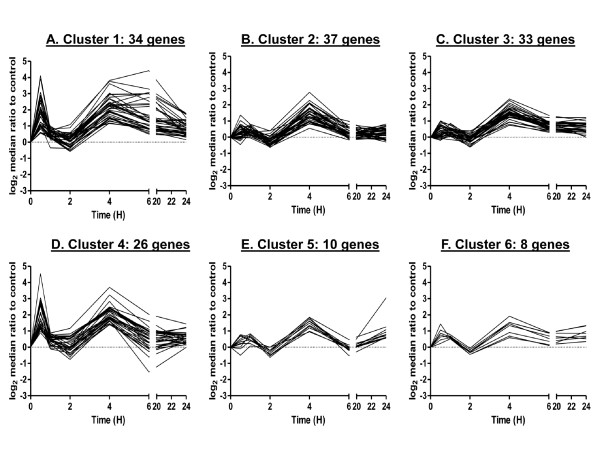
**STEM clustering on gene expression in the bystander response to 0.5 Gy α-particles**. Six significant clusters resulting from c = 3 and m = 100 are displayed as time course plots of median log_2 _gene expression ratios in bystander vs. non-irradiated controls. The number of genes/curves assigned to each cluster is shown. Data are medians across four independent biological replicates. Time is shown in hours (H).

In order to compare the two clustering methods on a related cellular response, we applied STEM and FBPA to gene expression curves after bystander exposure to radiation. We discuss the results of clustering bystander responding genes using the STEM platform first. We selected the results from c = 3 and m = 100 for analysis of bystander gene expression. Again, results were relatively consistent across input parameters. These parameters resulted in significant clustering of 160 out of the 238 cases (67.2%). Figure 5 shows the gene expression profiles for the most significant clusters, 6 out of 100 possible clusters. The number of genes included in each cluster was again relatively uniform, ranging from 8 genes in Cluster 6 to 39 genes in Cluster 1 (Figure 3C). Although the results visually showed good cluster tightness, we noted that Clusters 2, 3, 5 and 6 looked relatively similar, suggesting that these clusters represented subdivisions of a larger cluster, limiting the usefulness of the results, despite the use of 100 distinct profiles. Additional file 4 lists clustered genes from the application of STEM to the bystander gene response.

The expression curves of the 238 genes in bystander cells were also clustered using FBPA. Again, to determine the optimal number of clusters, we used the gap statistic. We examined k = 3 and 5, which both showed near zero inequalities. Average homogeneity was found to be 2.376 and average silhouette was 0.372 for k = 5 (Table 3). For k = 3, average homogeneity was 2.950 and average silhouette, 0.489 (data not shown). Because reasonable structure and good tightness were found with k = 5, we chose to present this clustering. The Rand index to the manually curated clustering was 0.745 (Table 1), indicating high similarity equivalent to that of STEM. Additional file 5 lists clustered genes from the application of FBPA to the bystander gene response.

**Table 3:** FBPA cluster statistics on gene expression in bystander cells

Cluster	Cardinality	Homogeneity	Average Silhouette
1	107	2.301	0.205
2	71	1.681	0.416
3	33	3.953	0.743
4	24	2.681	0.412
5	3	1.733	0.857
Total	238	2.376	0.372

Cluster cardinality is the number of genes in each cluster, homogeneity and average silhouette measurements are described in the Methods

**Figure 6:**
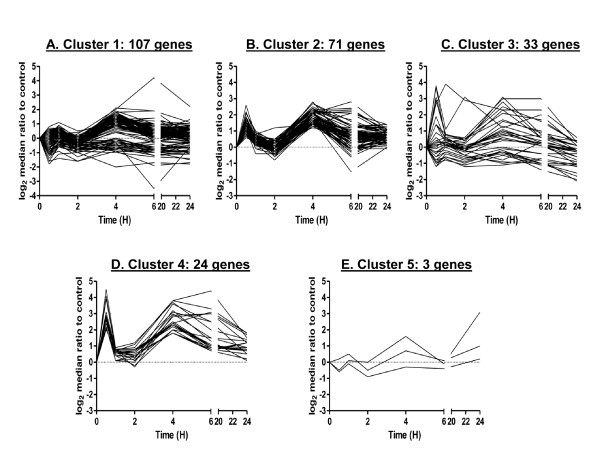
**FBPA clustering on gene expression in the bystander response to 0.5 Gy α-particles**. The five clusters resulting from k = 5, where k is cluster number, are displayed as time course plots of median log_2 _gene expression ratios in bystander vs. non-irradiated controls. The number of genes/curves in each cluster is indicated. Data are medians across four independent biological replicates. Time is shown in hours (H).

The FBPA clusters are shown in Figure 6. The within-method metrics indicate that Clusters 2 and 5 showed homogeneity and Clusters 3 and 5 showed good separation in terms of average silhouette (Table 3). As with the FBPA clustering of radiation-responsive genes, the bystander genes were not uniformly distributed across clusters: 45% of genes were assigned to Cluster 1, 30% to Cluster 2, 14% to Cluster 3, 10% to Cluster 4 and 1.3% to Cluster 5 (Figure 6).

Comparing the bystander FBPA clusters to STEM clusters, STEM Cluster 1 mapped well to FBPA Cluster 2 (71.9% match). STEM Clusters 2, 3, and 5 mapped relatively well to FBPA Cluster 1 (62.2%, 72.7% and 60%, respectively). As noted above, many of the gene expression curves assigned to STEM Clusters 2, 3, 5 and 6 showed a generally similar pattern. STEM Cluster 6, however, mapped most closely to FBPA Cluster 2 (50% match). STEM Cluster 4 mapped partially to FBPA Clusters 2 and 4 (48.4% and 38.7% respectively), while FBPA Clusters 3 and 5 did not match any of the STEM clusters well.

### Between Method Agreement

After performing clustering on the microarray and qRT-PCR data using the STEM software and the FBPA approach, we used the Rand index to compare the agreement of methods. The Rand index table (Table 1) indicates this was generally good across clusterings. We note higher consistency between FBPA clusterings of the data (0.681, 0.742) than STEM clusterings of the data (0.644, 0.490) in both irradiated and bystander conditions. Both the STEM and FBPA methods showed lower agreement with the manually curated standard for qRT-PCR data than for microarray data as shown in the first row of Table 1, but the STEM clustering performed noticeably more poorly (0.607, 0.661 vs. 0.486, 0.483). As all clustering methods indicated relatively good clustering agreements, we next examined the biological enrichment of individual clusters to explore the usefulness of the information generated by clustering genes by patterns.

### Network and ontology analysis for direct irradiation gene response

We next analyzed individual clusters using biology-based approaches that facilitate understanding biologically relevant responses. The first approach was an ontology-based analysis using the PANTHER database [33]. We first considered STEM clustering of the irradiation gene response. As mentioned previously, STEM clustering provided six significant clusters with relatively uniform cardinality (Figure 3A). We applied gene ontology methods using the PANTHER web-based tool to assess the biological relevance of these six clusters. We started by mapping genes in each cluster to functional and pathway annotations in PANTHER. This step maps gene identifiers to annotations in the PANTHER database and is important because of redundancy of biological annotations in databases, which may affect the outcome of analyses [34,35]. We found that coverage of mapping in the six clusters was randomly spread from 67% in the largest cluster, Cluster 1, to 93% mapped genes in Cluster 2 (Figure 3A). Surprisingly, gene ontology enrichment showed that only Cluster 3 was significantly enriched for biological processes, which spanned diverse functions from apoptosis to cell signaling and proliferation (Table 4). Minimal biological structure was apparent in the other clusters. Network analyses on the individual STEM clusters implicated p53 as a transcriptional regulator in all clusters except those containing genes down-regulated at 4 hours (not shown). There was no significant correlation between mapping coverage of genes in STEM clusters (Figure 3A) and functional categorization (Table 4).

**Table 4:** PANTHER gene ontology analysis on STEM clustering of gene expression data after direct irradiation

**Biological Process**	**Cluster**					
	
	**1**	**2**	**3**	**4**	**5**	**6**
	
Intracellular signaling cascade	NS	NS	1.03 × 10^-6 ^ (10)	NS	NS	NS
Cytokine and chemokine mediated signaling pathway	NS	NS	1.04 × 10^-6 ^ (7)	NS	NS	NS
Apoptosis	NS	NS	1.25 × 10^-6 ^ (8)	NS	NS	NS
Ligand-mediated signaling	NS	NS	1.34 × 10^-6 ^ (8)	NS	NS	NS
NF-kappaB cascade	NS	NS	2.10 × 10^-6 ^ (5)	NS	NS	NS
Immunity and defense	NS	NS	1.07 × 10^-5 ^ (10)	NS	NS	NS
Signal transduction	4.09 × 10^-2 ^ (9)	NS	3.63 × 10^-5 ^ (14)	NS	4.09 × 10^-2 ^ (9)	NS
Macrophage-mediated immunity	NS	NS	4.38 × 10^-5 ^ (5)	NS	NS	NS
Cell proliferation and differentiation	NS	NS	1.79 × 10^-4 ^ (8)	NS	NS	NS
MAPKKK cascade	NS	NS	2.10 × 10^-4 ^ (5)	NS	NS	NS
Cell communication	NS	NS	2.75 × 10^-4 ^ (9)	NS	NS	NS
Cell surface receptor mediated signal transduction	NS	NS	3.62 × 10^-4 ^ (10)	NS	4.79 × 10^-2 ^ (7)	NS
Inhibition of apoptosis	NS	NS	1.27 × 10^-3 ^ (4)	NS	NS	NS
Granulocyte-mediated immunity	NS	NS	5.75 × 10^-3 ^ (3)	NS	NS	NS
T-cell mediated immunity	NS	NS	6.42 × 10^-3 ^ (4)	NS	NS	NS
JNK cascade	NS	NS	6.82 × 10^-3 ^ (3)	NS	NS	NS
Cell cycle control	NS	NS	8.58 × 10^-3 ^ (5)	NS	NS	NS
JAK-STAT cascade	NS	NS	1.81 × 10^-2 ^ (3)	NS	NS	NS
Calcium mediated signaling	NS	NS	4.52 × 10^-2 ^ (3)	NS	NS	NS

Significant PANTHER biological processes categories, p-values are Bonferroni corrected, p-values < 0.05 are shown. The number of genes in each category is shown in parentheses. NS is Not Significant.

**Figure 7:**
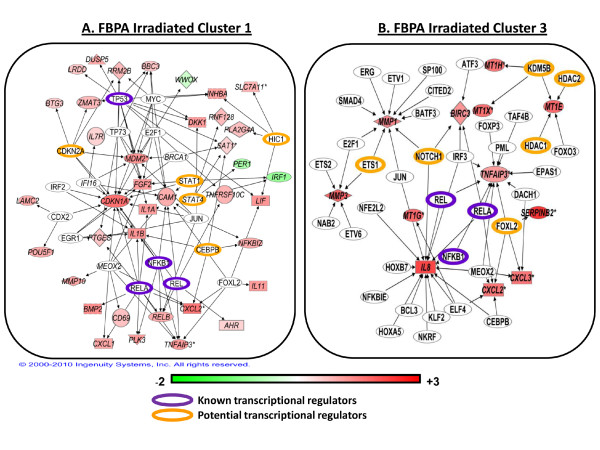
**Pathway analysis of FBPA clustering on gene expression after direct irradiation**. FBPA clusters 1 and 3 are shown here as networks generated by mapping genes using Agilent IDs and using the "grow" tool in IPA to identify potential transcriptional regulators upstream of genes in each cluster. Arrows indicate the direction of activity of a protein (empty node) interacting with the gene or gene product that is differentially expressed (colored node). Nodes are colored by log_2 _gene expression ratios in irradiated cells at 4 hours. On the scale bar, red indicates up-regulation and green, down-regulation of mRNA compared to sham-irradiated controls. Highlighted nodes indicate known transcriptional regulators involved in the radiation gene response in IMR-90 fibroblasts (violet outline) and potential new regulators of transcription (orange outline) suggested by the IPA analyses.

We then analyzed clusters from FBPA (k = 4) for the 238 directly irradiated gene expression curves (Figure 4). Again, we saw that there was no significant trend of mapping coverage across clusters. The largest cluster, Cluster 1, included 145 genes, 25% of which were unmapped in PANTHER (Figure 3B). In Table 5, we summarize the result of querying the PANTHER database for significant biological processes in each cluster in irradiated samples. Cluster 1 was significantly enriched in genes involved in cell cycle processes (p-value 10^3^) and Cluster 2 was significantly enriched in genes related to immunity and cell defense mechanisms (p-value 10^-3^). Network analysis suggested that these groups of genes are probably related or responsive to the p53 family of cell cycle regulators and to NF-κB transcriptional regulation, respectively (Figure 7A). Both these transcription factors are known to be major players in the gene expression response to irradiation. In Cluster 3 a group of genes belonging to immune cell-mediated immunity (p-value 10^-5^) and NF-κB cascade genes (p-value 10^-3^) were significantly clustered. Surprisingly, biological functions were clearly separated among the first three clusters, suggesting distinct biological functionality with only one significantly enriched biological process, NF-κB cascade, in common between Clusters 1 and 3. Generally, we found a cell signaling cluster, a cell cycle/cell death cluster, and a cell-mediated immunity cluster. Cluster 4, with only 6 genes, gave no significant results.

**Table 5:** PANTHER gene ontology analysis on FBPA clustering of gene expression data after direct irradiation

**Biological Process**	**Cluster**			
	
	**1**	**2**	**3**	**4**
	
Cell proliferation and differentiation	1.79 × 10^-3^ (14)	1.06 × 10^-2^ (8)	NS	NS
Signal transduction	3.41 × 10^-3^ (28)	3.28 × 10^-3^ (16)	NS	NS
Immunity and defense	6.95 × 10^-3^ (15)	5.34 × 10^-2^ (8)	NS	NS
Ligand-mediated signaling	9.81 × 10^-3^ (9)	NS	NS	NS
Cell surface receptor mediated signal transduction	1.11 × 10^-2^ (18)	2.22 × 10^-3^ (12)	NS	NS
Inhibition of apoptosis	2.48 × 10^-2^ (5)	NS	NS	NS
NF-kappaB cascade	3.96 × 10^-2^ (4)	NS	8.35 × 10^-3 ^ (3)	NS
Apoptosis	NS	1.04 × 10^-3^ (7)	NS	NS
Cell cycle control	NS	1.06 × 10^-3^ (7)	NS	NS
Cell cycle	NS	5.05 × 10^-2^ (7)	NS	NS
Granulocyte-mediated immunity	NS	NS	5.94 × 10^-5 ^ (4)	NS
Macrophage-mediated immunity	NS	NS	4.49 × 10^-2 ^ (3)	NS
Calcium mediated signaling	NS	NS	3.54 × 10^-2 ^ (3)	NS
Cell motility	NS	NS	4.53 × 10^-2 ^ (4)	NS

Significant PANTHER biological processes categories, p-values are Bonferroni corrected, p-values < 0.05 are shown. The number of genes in each category is shown in parentheses. NS is Not Significant.

We further analyzed Clusters 1 and 3 using network analysis (Figure 7) to discover transcriptional regulatory modules that could potentially explain the differing results for these two clusters. Cluster 1 genes (145 genes out of 238) were largely under putative transcriptional control of p53 (TP53) and related proteins (TP73, E2F1 and MYC). In the same cluster there were also genes predicted to be under regulation of NF-κB family members (RELA, REL and NFKB1), Figure 7A. Visual assessment of Cluster 1 genes showed that this cluster included both biphasic responding genes and the single 4-hour peak genes (Figure 4A). Therefore, the finding through gene ontology and network analysis that this cluster combines both cell cycle and inflammatory responses might have been expected.

In contrast, in Cluster 3, analysis by gene ontology excluded cell cycle and apoptosis biology, but NF-κB cascade and granulocyte/macrophage mediated immunity were over-represented categories. Network analysis of Cluster 3 (Figure 7B) further substantiates the role of NF-κB family members (RELA, REL and NFKB1). This was a smaller (31 genes out of 238) and visually tighter cluster (Figure 4C). A group of 8 metallothionein genes belonged to this smaller cluster, suggesting coordinate regulation of these genes over time (Additional File 6A).

Metallothioneins are modulators of metal toxicity and important mediators of oxidative damage with a specific role in radical scavenging after radiation exposure [36]. Studies on metallothionein-null mice after x-irradiation have also demonstrated a protective role for these proteins based on micronuclei induction in blood cells [37-39]. Although levels of metallothionein gene expression vary in different cell lines, constitutively high levels are often observed in cancer cell lines [40,41]. Metallothionein expression can be induced in response to metal exposure, interleukins, cytokines, and stresses including ionizing radiation [36,40,42]. Metallothionein genes are known to be regulated by many transcription factors, such as Metal-responsive Transcription Factor-1 (MTF1), which is essential for inducing all metallothioneins [36,43]. Other studies, however, have shown that metallothionein gene expression can be modulated by histone modifiers [44]. The position of this gene family on human chromosome 16q13, which contains the 17 out of 18 metallothionein 1(*MT1*) gene iso-forms, in addition to *MT2, MT3 *and *MT4 *genes [38], further substantiates a potential epigenetic control mechanism for MT gene expression. Our network analysis of the genes in Cluster 3 (Figure 7B) suggested that epigenetic regulation may also play a role in metallothionein gene expression, specifically through the histone modifiers KDM5B, HDAC1 and HDAC2.

KDM5B (JARID1B), which can act as a transcriptional repressor by de-methylating histone H3 lysine residues bound to promoters [44], has been shown to be up-regulated by hypoxia stress in a HIF1α-dependent manner [45], although there are no previous reports of its response to ionizing radiation. Scibetta et al. [44] carried out extensive functional analyses of KDM5B and its effects on gene expression, and found *MT1E, MT1F, MT1 H *and *MT1L *mRNAs to be highly responsive to levels of KDM5B. Overexpression of KDM5B was shown to repress gene expression and RNAi-mediated knockdown of KDM5B increased expression of all the above metallothionein genes.

Histone deacetylases (HDAC1 and HDAC2), have also been shown to regulate metallothionein gene expression [46,47]. The HDAC proteins act as transcriptional repressors by de-acetylating histones and silencing chromatin. The direct effects of ionizing radiation on HDAC levels are not clearly known, but HDAC inhibitors are widely studied as radio-sensitizers of cancer cells [48]. Also, HDAC1 has been shown to interact directly with the KDM5B protein [41], raising the possibility that both proteins may act in concert to modulate the early response to radiation.

**Figure 8:**
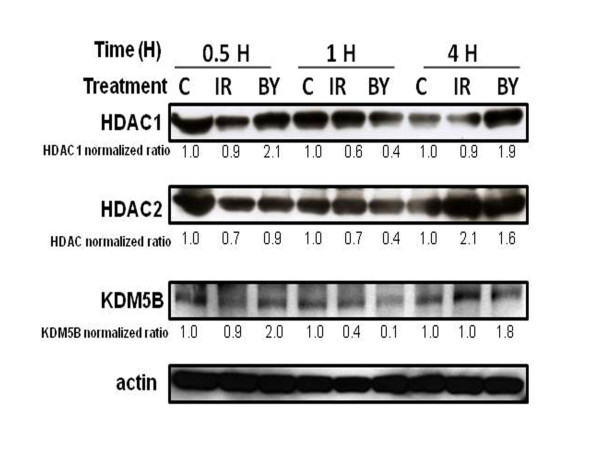
**Potential epigenetic regulators of metallothionein gene expression**. Western analysis of C (control), IR (directly irradiated) and BY (bystander) samples at 0.5 hr, 1 hr and 4 hr after treatment. Detected proteins were HDAC1, HDAC2, KDM5B and actin. Protein expression was measured by semi-quantitative densitometry and ratios relative to controls are shown for each lane after normalization to the actin internal control.

Using western blot analysis, we found that protein levels of KDM5B, HDAC1 and HDAC2 were all decreased an hour after exposure (Figure 8), preceding the 4-hour peak in metallothionein gene expression. These findings support the possible involvement of chromatin-level modifications in regulating gene expression in both directly irradiated and bystander cells. Histone deacetylation (by HDACs) and histone lysine demethylation (by KDM5B) activities could also potentially contribute to the responses observed for other genes in addition to the metallothioneins, suggesting coordinate epigenetic control of gene expression as an important component of the cellular radiation response.

The participation of trans-activating factors, such as transcription factors and co-activators that affect gene expression at promoter regions, in the radiation response is well known. However, the potential contributions of DNA topology changes and other epigenetic effects exerted by non-coding RNA, DNA methylation and histone modification are not as well studied in the radiation response. There is some evidence for epigenetic mechanisms such as DNA hypo-methylation after radiation exposure [14] but little is known about target genes and their dynamics, except in the case of the *INK4A *locus [49]. Our study, by clustering genes with similar time course responses after radiation and bystander treatments, suggested a possible role for epigenetic regulation of metallothionein levels.

### Network and ontology analysis in bystander gene response

STEM clustering of the bystander data for the 238 genes yielded 6 significant clusters (Figure 5) with uniform cardinality (Figure 3C) as seen in the case of irradiation (Figure 3A). Using the same approach as before, we applied gene ontology methods using the PANTHER web-based tool to assess the biological relevance of these six clusters. First, we mapped the genes in each cluster to see if any of the statistically significant clusters had largely unmapped genes. We found that the mapping of each cluster, once again, was randomly spread from 67% mapped genes in Cluster 3 to 90% in Cluster 5. Gene ontology analyses (Table 6) of these clusters showed that Cluster 1 had over-represented categories related to signaling and defense. Cell cycle processes were not significantly enriched in any bystander clusters as they were after direct irradiation, but apoptosis was significantly enriched in Cluster 2. *FAS, TNFRSF10C, TNFRSF10B, MYBL1 *and *MDM2 *were gene members in this category. STEM clustering in bystanders suggested only one biologically significant cluster with minimal biological findings in other clusters. This suggests that although this method can group genes into visually tight patterns, the algorithm is "blind" to functionally related genes that could be clustered together with more descriptive features, such as those used in FBPA. Network analysis of the six clusters confirmed that p53 and NF-κB family members were potential upstream regulators of gene expression in most of the STEM bystander clusters.

**Table 6:** PANTHER gene ontology analysis on STEM clustering of gene expression data in the bystander response

**Biological Process**	**Cluster**					
	
	**1**	**2**	**3**	**4**	**5**	**6**
	
Ligand-mediated signaling	1.33 × 10^-10 ^ (11)	NS	NS	NS	NS	NS
Cell communication	4.86 × 10^-7 ^ (12)	NS	NS	NS	NS	NS
Immunity and defense	3.24 × 10^-6 ^ (11)	NS	NS	NS	NS	NS
NF-kappaB cascade	3.77 × 10^-6 ^ (5)	NS	NS	NS	NS	NS
Cytokine and chemokine mediated signaling pathway	7.32 × 10^-5 ^ (6)	NS	NS	NS	NS	NS
Macrophage-mediated immunity	7.80 × 10^-5 ^ (5)	NS	NS	NS	NS	NS
Granulocyte-mediated immunity	1.31 × 10^-4 ^ (4)	NS	NS	NS	NS	NS
Cell proliferation and differentiation	4.42 × 10^-4 ^ (8)	NS	NS	NS	NS	NS
Apoptosis	8.07 × 10^-4 ^ (6)	6.76 × 10^-3 ^ (5)	NS	NS	NS	NS
Signal transduction	1.14 × 10^-3 ^ (13)	NS	4.09 × 10^-2 ^ (9)	NS	NS	NS
Intracellular signaling cascade	6.43 × 10^-3 ^ (7)	NS	NS	NS	NS	NS
Cell surface receptor mediated signal transduction	8.46 × 10^-3 ^ (9)	NS	NS	NS	NS	NS
T-cell mediated immunity	1.00 × 10^-2 ^ (4)	NS	NS	NS	NS	NS

Significant PANTHER biological processes categories, p-values are Bonferroni corrected, p-values < 0.05 are shown. The number of genes in each category is shown in parentheses. NS is Not Significant.

We also applied the same analyses to the FBPA (k = 5) clusters of the 238 bystander gene profiles (Figure 6). Again we observed no significant trend of mapping across clusters and the largest cluster, Cluster 1 with 107 genes, showed 28% of genes were unmapped in PANTHER (Figure 3D). A surprising result of gene ontology analysis was that there were no significantly enriched biological processes in Cluster 1 (Table 7), which grouped the most genes (107 genes out of 238). However, significant enrichment of biological processes was identified in Clusters 2, 3 and 4. Cluster 2 shared categories in common with Clusters 3 and 4; the most significant process in Cluster 2 was the NF-κB cascade (p-value 10^-6^). In Cluster 2, which was visually a tight cluster by pattern and magnitude of change (Figure 6B), other over-represented categories included signal transduction (p-value 10^-3^) and cell surface mediated signaling processes (p-value 10^-3^), both of which were also significantly enriched in Cluster 3. The most over-represented processes in Cluster 4 genes were granulocyte mediated immunity (p-value 10^-5^), NF-κB (p-value 10^-3^) and cytokine and chemokine signaling (p-value 10^-3^).

**Table 7:** PANTHER gene ontology analysis on FBPA clustering of gene expression data in the bystander response

**Biological Process**	**Cluster**				
	
	**1**	**2**	**3**	**4**	**5**
	
NF-kappaB cascade	NS	1.34 × 10^-6 ^ (6)	NS	4.74 × 10^-3 ^ (3)	NS
Ligand-mediated signaling	NS	1.72 × 10^-6 ^ (10)	NS	3.10 × 10^-3 ^ (5)	NS
Immunity and defense	NS	4.96 × 10^-6 ^ (14)	NS	1.24 × 10^-2 ^ (6)	NS
Cytokine and chemokine mediated signaling pathway	NS	6.82 × 10^-6 ^ (8)	NS	8.15 × 10^-3 ^ (4)	NS
Signal transduction	NS	1.86 × 10^-5 ^ (21)	2.51 × 10^-3 ^ (12)	NS	NS
Cell surface receptor mediated signal transduction	NS	3.13 × 10^-4 ^ (14)	4.45 × 10^-3 ^ (9)	NS	NS
Cell communication	NS	4.45 × 10^-4 ^ (12)	NS	3.70 × 10^-2 ^ (6)	NS
Cell proliferation and differentiation	NS	8.38 × 10^-4 ^ (10)	NS	NS	NS
Granulocyte-mediated immunity	NS	1.22 × 10^-3 ^ (4)	NS	2.73 × 10^-5 ^ (4)	NS
Macrophage-mediated immunity	NS	1.26 × 10^-3 ^ (5)	NS	2.57 × 10^-2 ^ (3)	NS
T-cell mediated immunity	NS	5.96 × 10^-3 ^ (5)	NS	NS	NS
Intracellular signaling cascade	NS	6.58 × 10^-3 ^ (9)	NS	NS	NS
Apoptosis	NS	1.86 × 10^-2 ^ (6)	NS	NS	NS
Inhibition of apoptosis	NS	NS	NS	1.98 × 10^-2 ^ (3)	NS
Calcium mediated signaling	NS	NS	NS	2.02 × 10^-2 ^ (3)	NS
Cell motility	NS	NS	NS	2.16 × 10^-2 ^ (4)	NS

Significant PANTHER biological processes categories, p-values are Bonferroni corrected, p-values < 0.05 are shown. The number of genes in each category is shown in parentheses. NS is Not Significant.

**Figure 9:**
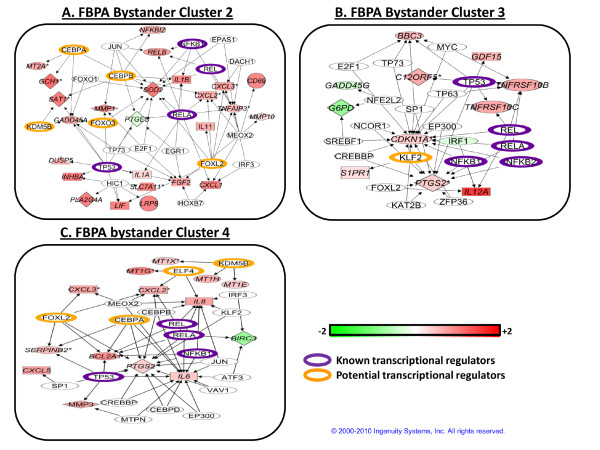
**Pathway analysis of FBPA clustering on gene expression in the bystander response**. FBPA clusters 2, 3 and 4 are shown here as networks generated by mapping genes using Agilent IDs and using the "grow" tool in IPA to identify potential transcriptional regulators upstream of genes in that cluster. Arrows indicate the direction of activity of a protein (empty node) interacting with the gene or gene product that is differentially regulated (colored node). Nodes are colored by log_2 _gene expression ratios in irradiated cells at 4 hours. On the scale bar, red indicates up-regulation and green, down-regulation of mRNA compared to sham-irradiated controls. Highlighted nodes indicate known transcriptional regulators involved in the radiation gene response in IMR-90 fibroblasts (violet outline) and potential new regulators of transcription (orange outline) suggested by the IPA analyses.

We further analyzed Clusters 2, 3 and 4 using network analysis to discover transcriptional regulatory modules that could potentially be responsible for coordinate regulation of these three clusters (Figure 9). We observed that in Clusters 2, 3 and 4 there were common hubs of transcriptional control. p53 and NF-κB proteins were potential transcription factors of genes in all three clusters, which had similar overall profiles (Figure 6). KDM5B/JARID1B was once again identified as a potential upstream regulator of genes in both Clusters 2 and 4 (Figure 9A and 9C). In Cluster 4, the genes potentially regulated by KDM5B were the same as those in FBPA Cluster 3 after irradiation (Figure 7B), and in bystanders KDM5B was also shown to be upstream of the *GADD45A *and *SAT1 *genes in Cluster 2 (Figure 9A). It was interesting that the metallothionein gene expression response in bystanders was similar to that in irradiated cells (Additional file 6B), suggesting that irradiated cells may be communicating a signal that induces epigenetic changes in both populations. Protein analysis on KDM5B, HDAC1 and HDAC2 levels showed that these histone modifiers are lowered in bystanders at 1-hour after treatment as in the directly irradiated cells (Figure 8). This suggests that the bystander metallothionein gene response maybe regulated similarly as in irradiated cells.

### Biological and statistical evaluation of clustering results

In terms of the clustering methodologies used here, the most surprising result was the high degree of biological information found using the FBPA clustering versus STEM clustering across both cell treatments, despite roughly equivalent computational evaluations. We observed similar enrichment results for other STEM clusterings of the data with various parameters (c = 1-3, m = 25-200, results not shown). Although there were some common processes between FBPA clusters, the gene ontology enrichment showed clear delineation of biological information. Related biological functions were focused in specific clusters, suggesting that features used in FBPA captured relevant biological details of the gene expression response curves. In radiation gene response, three out of four clusters gave distinct functional groups: a cell signaling cluster (Figure 4A, Table 5 and Figure 7A), a cell cycle/cell death cluster (Figure 4B, Table 5) and a cell-mediated immunity cluster (Figure 4C, Table 5 and Figure 7B). Network analysis clearly revealed the differences in individual players and suggested novel regulatory mechanisms for the coordinate responses.

By contrast, STEM resulted in only one cluster with biologically significant functions for both treatment conditions: irradiated Cluster 3 (Figure 2C and Table 4), and bystander Cluster 1 (Figure 5A and Table 6), which encompass processes from signal transduction modules (MAPKK and NF-κB), cell-specific immunity (granulocyte and macrophage), cell death and cell proliferation responses. The other STEM clusters appeared to have minimal enrichment of genes involved in a particular biological process, giving little direction for the inference of biological functions of the genes clustered, and also suggesting that STEM did not capture details relevant to co-regulated processes as well as FBPA did. We believe this is attributable to several factors. Firstly, we cite the use of biologically relevant features and dimension augmentation for FBPA clustering. Standard computational tools do not put the focus here and may ignore latent information in the data as a result. Secondly, FBPA is designed to be parsimonious. We used the gap statistic to identify possible clustering of the data, and we used within-method clustering metrics to assess and determine the number of clusters to be used. We put an emphasis on cluster separation, which was a good indicator of structure in the data. For example, in the case of the direct irradiation gene response, only STEM Cluster 3 was found to be significantly enriched for any biological functions, but STEM Clusters 1, 4, and 6 all mapped mainly to FBPA Cluster 1, suggesting that enrichment may have been missed because the STEM clusters were over-fitted to the data, forcing functionally related genes into separate clusters. As noted earlier, robust responses were expected following irradiation. Thus, parsimony in cluster number may be critical to grouping functionally similar genes. Thirdly, we consider the level of noise in the data. The STEM algorithm put an emphasis on visually tight clustering of the data over separation and parsimony. Raw expression information was used to discretize the data and typically a high number of candidate profiles were used to fit the data. Many of these candidate profiles and the genes assigned to them were determined to be insignificant as clusters. Thus, profiles that appear to be relative outliers were excluded and the resulting expression profiles were less noisy. In contrast, FBPA clustered every gene. This resulted in noisier clusters, but some of the "noise" may represent biologically relevant information, as we found here. Furthermore, some of the noise we see in the FBPA clustering may be the result of using gene expression profiles to display the clusters instead of the features to describe the gene expression curves (Figures 4, 6).

There were also consistencies between the clustering methods used. For example, cell cycle control processes were not over-represented in any clusters generated by FBPA or STEM in the bystander gene response, whereas, stress response, inflammation and cellular defense mechanisms were strongly implicated in the bystander gene expression response. Cell death, on the other hand, was a significant category in both STEM Clusters 1 and 2 (Table 6) and in FBPA Cluster 2 (Table 7) in bystanders. In the bystander gene response, there was more functional overlap between clusters compared with the radiation gene response (Table 7). In general, larger biological variation in gene expression was observed in bystanders, possibly due to the indirect nature of the signal and other factors such as cell culture conditions, confluence, temperature, etc. that can affect transmission of bystander signals. This may account for the result in bystander-FBPA Cluster 1 (the largest cluster, with 107 genes) where genes clustered together on the basis of features (Figure 6A) but did not belong to any significant biological process (Table 7, Cluster 1). Taking a closer look at putative regulators of genes that were clustered together suggested that in addition to the p53 and NF-κB pathways, there may be other players in the radiation response, which would not have been identified either by studying individual genes or by considering all the responding genes together as a single set.

## Conclusions

The objective of this study was to summarize and cluster time series gene expression in irradiated and bystander fibroblasts to uncover novel biologically relevant information. We applied a new clustering algorithm, FBPA, which used relevant features to cluster data. These features summarized the gene expression profiles and accounted for dependence over time. This method was devised specifically for sparse time series where model-fitting is not realistic. It is broadly applicable to other data sets. It does not require measurements to be taken at the same time points and can handle missing values. FBPA is scalable to a large number of genes, only restricted by processing capacity.

We compared FBPA to STEM, another popular clustering algorithm for short time series. While the two methods were comparable when using computational measures of evaluation, FBPA outperformed STEM in finding biologically meaningful clusters in both the irradiated and bystander cases. We believe this is because of the use of biologically relevant features that explain the data well and an emphasis on parsimony as opposed to strictly computational methods that do not address these factors.

Additionally, we compared the temporal response of mRNA to 0.5 Gy α-particle irradiation and in-contact neighboring bystander cells and confirmed trends in gene regulation. More interestingly, we were able to extract new information from the clustering results that predicted upstream regulators of gene expression not previously suggested by class comparison and ontology methods. Our analysis suggested a candidate novel gene regulatory mechanism involving histone modifications at promoter regions of metallothionein genes by KDM5B (lysine demethylase) and HDACs (histone deacetylases). Further studies on the role of these epigenetic mechanisms and the induction of metallothionein genes in response to α-particle irradiation will be required to understand the roles of these new players in the radiation response.

In conclusion, this study achieved the objective of extracting biological insights from quantitative data after grouping it into clusters and identifying novel processes in the precise regulation of individual biological molecules as a result of radiation. In this study, we addressed only mRNA level changes and it will be interesting to see if parallel measurements of "omic" data at other levels such as chromatin immunoprecipitation-array (ChIP-chip) information, proteomic and metabolomic data may be analyzed simultaneously using feature based clustering methods. Also, in this study we limited the analyses to genes shown to be differentially regulated at four hours, as a test set for the clustering methodology. We found that FBPA clustering can sort gene expression responses and subsequent biological enrichment of clusters can reveal new knowledge based on this sorting method. When this method is applied to the complete set of differentially regulated genes in the time series, it will also help us more fully understand the involvement of pathways that can affect cell and tissue integrity after exposure to radiation.

## Methods

### Cell culture, irradiation and RNA isolation

Early passage (population doubling < 35) IMR-90 human lung fibroblasts (Coriell repository, NJ) were sub-cultured in Dulbecco's modified Eagle's medium (Gibco) and Ham's F10 medium in a 1:1 mixture plus 15% fetal bovine serum. Mylar-bottomed culture dishes were prepared as described previously [50]. An inner dish with a base of 38-μm-thick Mylar strips was inserted into a larger dish with a 6-μm Mylar base. The 38-μm Mylar completely shields the α-particles so that only cells on the thinner Mylar areas of the dish were directly irradiated [50]. Cells seeded in these dishes formed a contiguous layer. Cells were exposed to 0 (sham irradiated) or 50 cGy ^4^He ions (125 keV per micron) as simulated α-particles using the track segment mode of the 5.5-MV Singletron accelerator at the Radiological Research Accelerator Facility of Columbia University. Four independent experiments were conducted, and each was performed in parallel with irradiated, bystander and sham-irradiated samples derived from a sub-cultivated pool of IMR-90 cells that were seeded from a single cryo-vial.

Directly irradiated (outer dish) and bystander (inner dish) cells were separated at 30 minutes, 1, 2, 4, 6 and 24 hours after exposure, and RNA was isolated from the exposed cultures and from time-matched sham-irradiated controls using Ribopure (Ambion, Life Technologies). All RNA samples had RNA integrity numbers >9.0 [51] and 260 nm/280 nm absorbance ratios >2.

### Microarray Data and Processing

Each sample was hybridized to an Agilent Whole Human Genome Oligo Microarray (G4112F) using the Agilent one-color workflow as previously described [9]. The extracted data from the time course microarrays were imported into BRB ArrayTools [52]. Genes were included if detected, as reported by gIsWellAboveBG (inclusive flag), which indicates if the spot expression measurement was greater than the background signal plus 2.6-fold of the standard deviation. Non-uniformity outliers were excluded using the gIsFeatNonUnifOL (exclusive flag). Genes for which more than 10% of the data was either not above background or was a non-uniformity outlier were filtered out. This resulted in a dataset of 72 microarray measurements of 25,280 genes. In order to preserve dependence across time points, the data were not normalized across arrays. Across-array normalization is known to modify the existing correlation structure [53] within a given dataset and, by extension, measurements made across time points. The full time-course microarray data are available through the Gene Expression Omnibus database using accession number GSE21059. Additional File 7 shows the processed (logarithmically transformed median expression ratios) data used for plotting cluster graphs for irradiated and bystander treatments.

Genes were selected for clustering based on 4-hour gene expression analyses performed in an earlier study [9]. In that study, 191 genes (FDR<0.10) showed differential expression in the irradiated vs. control at the 4-hour time point and 135 genes (FDR<0.10) were differentially expressed in the bystander vs. control, resulting in 253 unique gene features. With the addition of more time points, 15 of these probes did not pass the filtering criteria used here, leaving 238 features to be used in this analysis.

### Quantitative real time PCR (qRT-PCR) analysis

The High-Capacity cDNA Archive Kit (Life Technologies, Foster City, CA) was used to prepare cDNA from total RNA. A custom low-density TaqMan array (Life Technologies, Foster City, CA) was designed using validated assays. Gene expression assay information is in Additional File 8. 40 genes were selected for inclusion on the low-density array (LDA) on the basis of differential expression and low FDR, and seven endogenous control genes [9] were also included. Gene validation studies were carried out using the ABI 7900 Real Time PCR System as previously described [9].

Relative fold-inductions were calculated by the ΔΔC_T _method as described previously [54] using SDS version 2.3 software (Life Technologies). We applied geNorm [55] to the seven endogenous control genes on the LDAs to determine the most appropriate genes for normalizing the fold-change results. The LDA data were normalized to the geometric mean of peptidylprolyl isomerase A (*PPIA*) and ubiquitin C (*UBC*) gene expression levels. We used qRT-PCR measurements of 40 genes across the entire time course and used the median of ratios to control at each time point to generate heatmaps. BRB-ArrayTools was used to generate a heat map visualizing the median logarithmically transformed expression ratios for all four replicates generated by both microarray and qRT-PCR to compare gene expression across time and between measurement methods. qRT-PCR expression data are provided in Additional File 8.

### Clustering Microarray and PCR Data

We used two clustering methods to cluster the data. The STEM algorithm and software, described below, was developed by Ernst et al. [23]. We also proposed an approach using relevant features of the time course. Both methods are non-parametric forms of clustering, in the sense that they do not impose distributional or model-based assumptions on the data.

For the purpose of both clustering algorithms, expression measurements for a given gene, g, and replicate, r, for irradiated (*A*) and bystander (*B*) samples were represented as a function of control (*C*) expression, as *x_igr _= log_2_(A_igr_/C_igr_) *or *x_igr _= log_2_(B_igr_/C_igr_)*, where *i=*1,2,..., *n*, *n *is the number of time points used, *x_igr _*indicates the relative expression measurement for irradiated or bystander genes at the time point *i*, *A_igr _*is the unlogged expression in the irradiated sample at time point *i *and *B_igr _*is the unlogged expression in the bystander sample at time point *i*. We used *x_igr _*for both alpha and bystander expression here because the methods were agnostic to the particular treatment being considered. Representing the data as a ratio was possible because of the paired nature of the data. Irradiated data and bystander data were clustered separately for the microarray data but together for the smaller qRT-PCR data set.

### STEM method

First, we used the STEM (Short Time series Expression Miner) algorithm and software presented in [23] (Java implementation with a graphical user interface available from http://www.cs.cmu.edu/~jernst/st/). Briefly, a set of model profiles based on units of change, *c*, was defined. For example, if *c *= 2 then, between successive time points, a gene can go up either one or two units, stay the same, or go down one or two units. The clustering system may also define one unit differently for different genes. Thus, the number of possible profiles for n time points is (2*c*+1)^*n*-1^. From these possible expression profiles, a set of candidate profiles, size m, which was user-defined, were chosen such that the minimum distance between any two profiles was maximized. Each gene was assigned to the closest profile using a Pearson correlation based distance metric. To determine significance level for a given cluster, a permutation based test was used to quantify the expected number of genes that would be assigned to each profile if the data were generated at random. Therefore, while all genes were clustered, not every gene was in a significant cluster.

Inputs to the algorithm were the logged median expression for each gene and the parameters, *c *and *m*, discussed above. The logged median expression for a given gene was defined as $u_{i}=\underset{r}{med}\;x_{igr}$, where *i *= 1,2,..., *n*, *n *is the number of time points, *r *= 1,2, ..., *R*, *R *is the number of replicates, *x_igr _*is the expression at time point *i *for gene *g *and replicate *r *. We selected the median expression over the replicates rather than the mean because it was more robust to outliers. We examined results for *c *= 1 to 3 and *m *= 25 to 200 for both irradiated and bystander data, results were similar across clusterings.

### Features-Based PAM Algorithm (FBPA)

We now provide a description of the FBPA clustering method. An extended comparison of FBPA with other time course analyses methods can be found in [27], where we also describe the performance of FBPA on other real data sets as well as simulated data sets. As a first step, characteristics of the data were summarized in a number of well-chosen features: slopes between adjacent time points, maximum and minimum expression ratios, time of maximum and minimum expression, and steepest positive and negative slope, for a total of 12 features. Selection of these features represented our goal of being able to understand and describe profiles of expression over time.

#### Slope between adjacent time points

The slope was chosen as a feature because it is a measure of the change in expression over time, and is a first order approximation of the shape of the curve or gene expression profile. To calculate this we appended an initial measurement of zero to the expression and time for each replicate to capture the initial slope. We then calculated the median slope between each pair of adjacent time points. For a given gene, *g*, we created a vector of median slopes, *v*, for each profile as $v_{ig}=\underset{r}{med}\left(\frac{x_{(i+1)gr}-x_{igr}}{t_{i+1}-t_{i}}\right)$, where *i *= 1,2,..., *n*-1, *n *is the number of time points, *r *= 1,2,..., *R*, *R *is the number of replicates, *x_igr _*is the expression at time point *i *for gene *g *and replicate *r *and *t *is the time at time point *i*. Thus, for *n *time points, there were *n-1 *distinct slopes.

#### Maximum and minimum expression ratios

The maximum and minimum expression ratios were important for finding genes with the same magnitude of expression. Biologically, maximum and minimum expression ratios reflected the impact of signaling via specific transduction pathways and represented the best window of measurement of this change [56]. These measurements were found from the median ratios over all replicates for a given gene across time points. The maximum expression for a given gene was defined as $\underset{i}{\max}\;\underset{r}{med}\;x_{igr}$ and the minimum expression was defined as $\underset{i}{\min}\;\underset{r}{med}x_{igr}$, where *i *= 1,2,..., *n*, *n *is the number of time points, *r *= 1,2,..., *R*, *R *is the number of replicates, *x_igr _*is the expression at time point *i *for gene *g *and replicate *r*.

#### Time to maximum and time to minimum expression

Time to minimum and maximum expression and slope between measurements reflect the dynamics of individual gene expression and in many cases where common patterns are observed indicate coordinate control of transcription rates of a group of genes by a common transcription factor [56]. The time of maximum expression for a given gene was defined as the *i *corresponding to $\underset{i}{\max}\underset{r}{med}x_{igr}$ and the minimum expression was defined as the *i *corresponding to $\underset{i}{\min}\underset{r}{med}x_{igr}$, where *i *= 1,2,..., *n*, *n *is the number of time points, *r *= 1,2,..., *R*, *R *is the number of replicates, *x_igr _*is the expression at time point *i *for gene *g *and replicate *r*.

#### Steepest positive and steepest negative slopes

The steepest positive and negative slopes indicate the maximum rate of over-expression and under-expression. This feature was selected because it emphasizes these extreme rate changes. The measurements were defined using the median slope as described above and taking the maximum positive slope and the maximum negative slope. Thus, the steepest positive slope for a given gene was defined as $\underset{i}{\max}v_{i}$ and the steepest negative slope was defined as $\underset{i}{\max}(-v_{i})$, where *i *= 1,2,..., *n*-1, *n *is the number of time points, *v *is the slope between time point *i *and *i+*1.

Following this, we used the PAM algorithm [28] to cluster the data. Inputs to the algorithm were all of the features described above with equal weight on each. Euclidean distance was used to measure dissimilarity among the selected features.

The number of clusters, k, was determined via the gap statistic [31]. Here, we examined the gap from *k *= 3-15 for both irradiated and bystander conditions. The number of clusters *k *is generally chosen where gap(*k*)>gap(*k*+1)-s*_k _*and s*_k _*is the estimate of standard deviation from the gap. However, we examined all "elbow points" on the graphs and presented those that provide the best results in terms of separation of clusters and the homogeneity metric.

### Evaluating clustering methods

In general, cluster validity can be assessed on three bases: within method metrics, between-method metrics and cluster significance [57]. First, within-method metrics were used to validate cluster quality. By definition, objects within a given cluster were assumed to be similar, while those in different clusters were dissimilar. In FBPA, we used within-method clustering metrics to measure cluster homogeneity and separation. Because the STEM algorithm obfuscated its derived gene profiles, this was not possible for the STEM clustering. Homogeneity is a metric that measures the amount of variation within clusters, showing the tightness of the cluster. It is defined as the average distance of an element to its cluster center over all data points: $H_{ave}=\frac{1}{P}\sum_{i=1}^{P}D\left(g_{i},F(g_{i})\right)$ where *P *is the total number of genes in the cluster *D *is a distance function, g_i _is the ith gene and *F(g_i_) *is the cluster centroid for *g_i_*. Thus, the closer *H_ave _*is to zero the tighter the clustering is. We used Euclidean distance for *D*. However, the scale of "good" and "bad" were difficult to determine. Here we took measurements greater than three as showing poor homogeneity and measurements less than two as showing good homogeneity. To measure separation, we used the average silhouette [58]. First, an individual silhouette, *s(i)*, ranging from -1 to 1 was measured for each gene. This measured the average distance to all the elements in its assigned cluster and compared it to that of the closest cluster. An average silhouette width over 0.5 suggested a strong structure, 0.25-0.5 suggested a reasonable structure, and <0.25 suggested no substantial structure.

Second, between-method metrics were used to evaluate cluster agreement. Here, we validated findings between the two methods as well as between each method and manually curated clustering. The Rand index [7] was used to measure similarity of the two clustering algorithms; it ranged from 0 to 1 and the closer to 1, the more similar the two clustering algorithms are. However, this index approaches 1 as the number of clusters increases. Other options are also possible [59,60].

Third, cluster significance methods focus on the likelihood that the cluster structure has not been formed by chance. A fundamental difference between the above two clustering algorithms was that STEM pre-determines cluster patterns and, while it assigned all genes to clusters, it only designated some clusters as significant. Cluster significance was determined by a permutation based test, used to quantify the expected number of genes that would be assigned to each profile if the data were generated at random. In this way, the STEM algorithm measured cluster likelihood. We did not provide this for FBPA. The within-method silhouette and homogeneity metrics allowed us to look "under the hood" at individual clusters and make inferences on them. Given the caveat that these validation metrics are guidelines, ultimately subject to biological validation of patterns in gene expression, we felt that this approach was reasonable in the exploratory data analysis framework. It is also worth mentioning here that the significant clusters determined by STEM did not necessarily imply biologically significant clusters.

### Validation of clustering on qRT-PCR measurements

We used qRT-PCR confirmed genes as a smaller subset of genes to assess between method clustering. Because of the small number of genes used, the 80 irradiated and bystander curves were clustered together. After examining results for various parameter combinations using STEM, we found that results were relatively consistent around the choice of c. Smaller values of c resulted in fewer genes being clustered. Thus, we selected c = 3 and m = 25 for further analysis. This run clustered 57 out of the 80 cases (71%). The Rand Index to the manually curated clustering was 0.486 for the directly irradiated cases and 0.483 for the bystander cases, indicating average similarity to the manually curated standard. Here we see the STEM algorithm shows more noise. This is potentially because we chose a higher value for the units of change (c = 3) but a lower number of pre-defined profiles (m = 25). We did this to significantly cluster more genes, but the cost is higher noise in the resulting profiles. Nevertheless, the clusters did show distinct patterns.

To confirm results, we also clustered the median expression curves generated by qRT-PCR using FBPA. Again, because of the small number of genes confirmed by PCR, we clustered irradiated and bystander genes together and used the results to measure agreement only. Using the gap statistic method and plot, we examined k = 3 and k = 8 further. Based on within-method evaluation, we determined to use 8 clusters, which showed both better separation in terms of the average silhouette and better homogeneity. For k = 3, the average homogeneity was 3.969 and the average silhouette was 0.385. For k = 8, we had an average homogeneity of 2.345 and an average silhouette of 0.402. Because reasonable structure was found with k = 8, we chose this clustering. The Rand Index to the manually curated standard was 0.607 for the directly irradiated cases and 0.661 for the bystander cases, indicating good similarity.

### Gene ontology and pathway analysis

Following the separate clustering analysis of irradiated and bystander gene expression curves, we imported the gene sets from each cluster into PANTHER [33]. The genes/proteins in each list were mapped, and then functionally annotated and searched for significant functional enrichment using the PANTHER pathways and biological processes categories. Categories with a Bonferroni-corrected p-value less than 0.05, as calculated by the PANTHER software, were considered significant.

The sets of genes after clustering were also separately imported into Ingenuity Pathways Analysis (IPA) (Ingenuity^® ^Systems, http://www.ingenuity.com) to analyze network interactions between the genes. We applied pathway analysis as a complementary method of biological analysis of the gene groups generated by clustering. This approach allowed us to visualize potential interactions between the members of clusters, and to look for common upstream regulators. We applied very specific criteria, limiting our analyses to relationship type "expression/transcription" and molecule type "only upstream transcriptional regulators of genes," to each cluster of genes one by one. In clusters dominated by down-regulated genes, we also queried potential coordinated targeting by microRNA species that can suppress mRNA levels of more than one gene.

### Western Blots

For protein isolation directly irradiated (outer dish) and bystander (inner dish) cells were separated and trypsinized at specified times (30 minutes, 1 hour and 4 hours) after irradiation. Cells were collected, washed and lysed in 25% glycerol, 40 mM HEPES at pH 7.5, 1 mM DTT, 0.35 M NaCl, 0.5% NP-40 and Protease inhibitor mixture (HALT, Thermo Scientific). Protein concentrations were determined using the bicinchoninic acid method (Pierce) and measured using the Nanodrop-1000 spectrophotometer (Thermo Scientific). 50 micrograms of protein was used for western analysis and separated on 4-12% Tris-Glycine gradient polyacrylamide gels (Invitrogen, cat#EC6035BOX). Primary antibodies were from Abcam: HDAC1 (cat # ab46985), HDAC2 (cat# ab12169), and KDM5B (cat# ab50958) and from Chemicon: actin (cat# mab 1501). Secondary antibodies were conjugated to horseradish peroxidase and signals were detected using enhanced chemi-luminescence (Amersham, GE). Relevant bands were quantified by densitometry using Image J (NIH, http://rsb.info.nih.gov/ij/), background corrected and normalized to actin levels, then compared to time matched controls.

## List of abbreviations

FBPA: Feature based portioning around medoids algorithm; STEM: Short time series expression miner; NF-κB: Nuclear factor kappa B; HDAC: Histone deacetylases; BEIR: Biological Effects of Ionizing Radiation; PANTHER: Protein analysis through evolutionary relationships; qRT-PCR: quantitative reverse-transcription and polymerase chain reaction; GEO: Gene expression omnibus; FDR: False discovery rate; GO: Gene ontology;

## Authors' contributions

SAG and AS drafted the manuscript. SAG designed the study and performed all biological experiments and analyses. MM and AS carried out the cluster analyses and developed the supporting statistical methods. SAA and MM conceived of the study and participated in the design of experiments and the writing of the manuscript. All authors read and approved the final manuscript.

## Supplementary Material

Additional file 1 **Manually curated clustering, pdf file**. Click here for file

Additional file 2 **STEM clustering on irradiation gene response, MS excel file**. Click here for file

Additional file 3 **FBPA clustering on irradiation gene response, MS excel file**. Click here for file

Additional file 4 **STEM clustering on bystander gene response, MS excel file**. Click here for file

Additional file 5 **FBPA clustering on bystander gene response, MS excel file**. Click here for file

Additional File 6 **Metallothionein expression levels in irradiated and bystander cells, pdf**. Click here for file

Additional file 7 **Microarray data from irradiated and bystander cells, MS excel file**. Click here for file

Additional file 8 **qRT-PCR data from irradiated and bystander cells, MS excel file**. Click here for file
