# Supplementary material for: Time-series clustering of gene expression in irradiated and bystander fibroblasts: an application of FBPA clustering
Source: BMC Genomics. 2011 Jan 4;12:2. doi: 10.1186/1471-2164-12-2 (PMC3022823; doi:10.1186/1471-2164-12-2)

Additional File 1. Manually curated clustering

(1) no early peak; no change; (2) 2 peaks, 2 dips; (3) 2 peaks and 2 dips with a shallow second dip; (4) 2 peaks and 1 dip; (5) 2 peaks, 1 dip with a low magnitude first peak (6) 2 peaks and 1 dip with a high magnitude first peak and (7) down at 4 hours.

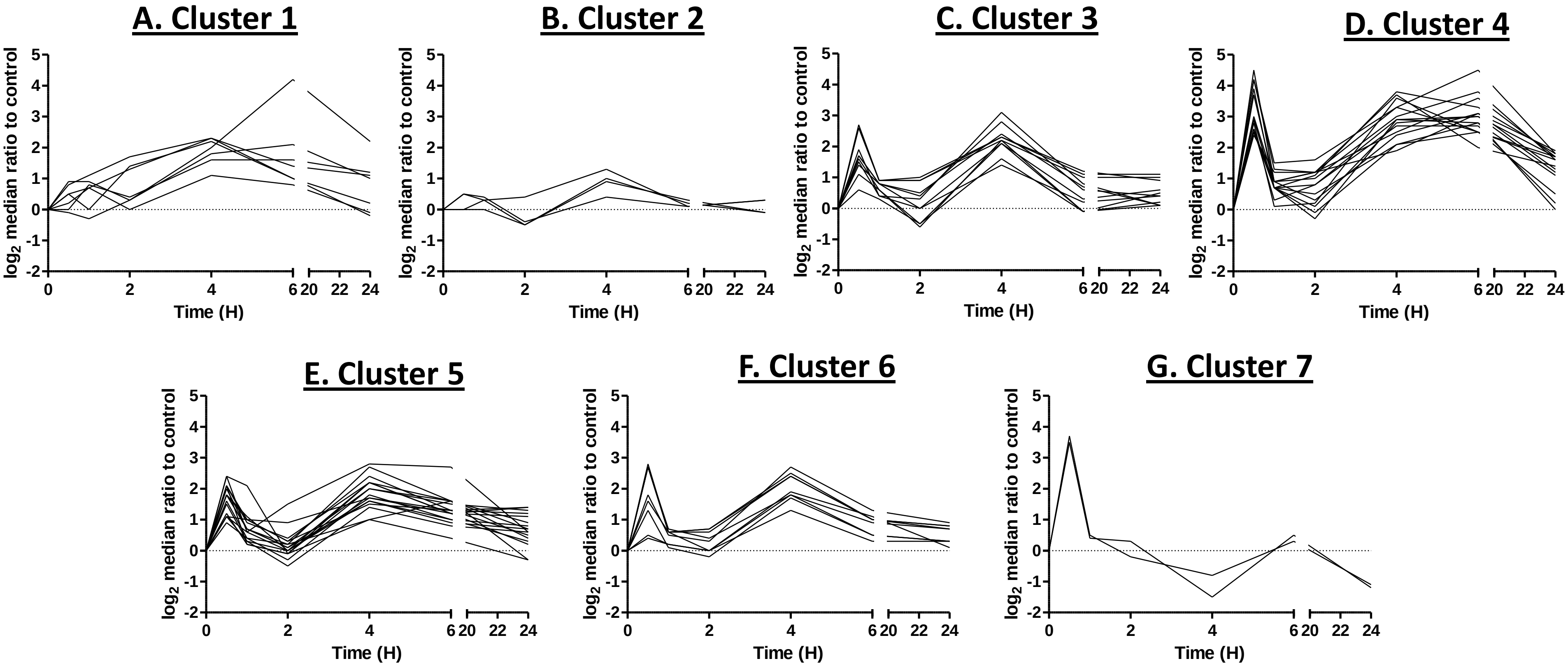

Supplement: Additional file 1 — Manually curated clustering, pdf file. [file 1471-2164-12-2-S1.PDF]
