# Supplementary material for: Time-series clustering of gene expression in irradiated and bystander fibroblasts: an application of FBPA clustering
Source: BMC Genomics. 2011 Jan 4;12:2. doi: 10.1186/1471-2164-12-2 (PMC3022823; doi:10.1186/1471-2164-12-2)
Supplement: Additional File 6 — Metallothionein expression levels in irradiated and bystander cells, pdf. [file 1471-2164-12-2-S6.PDF]

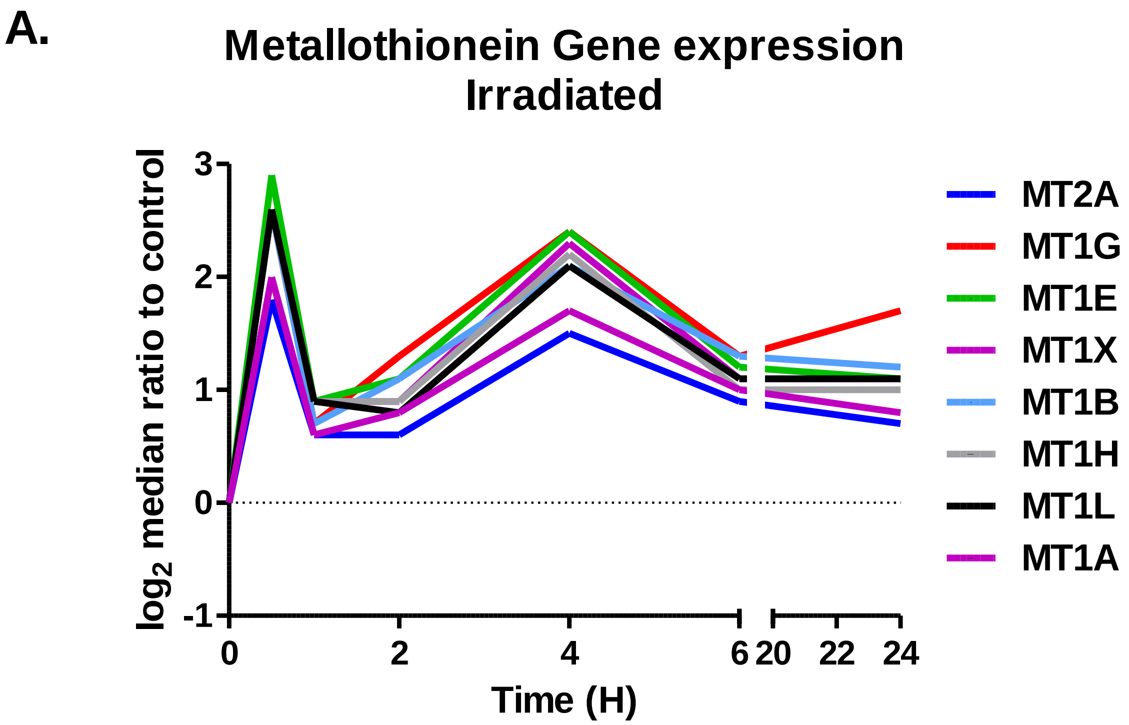

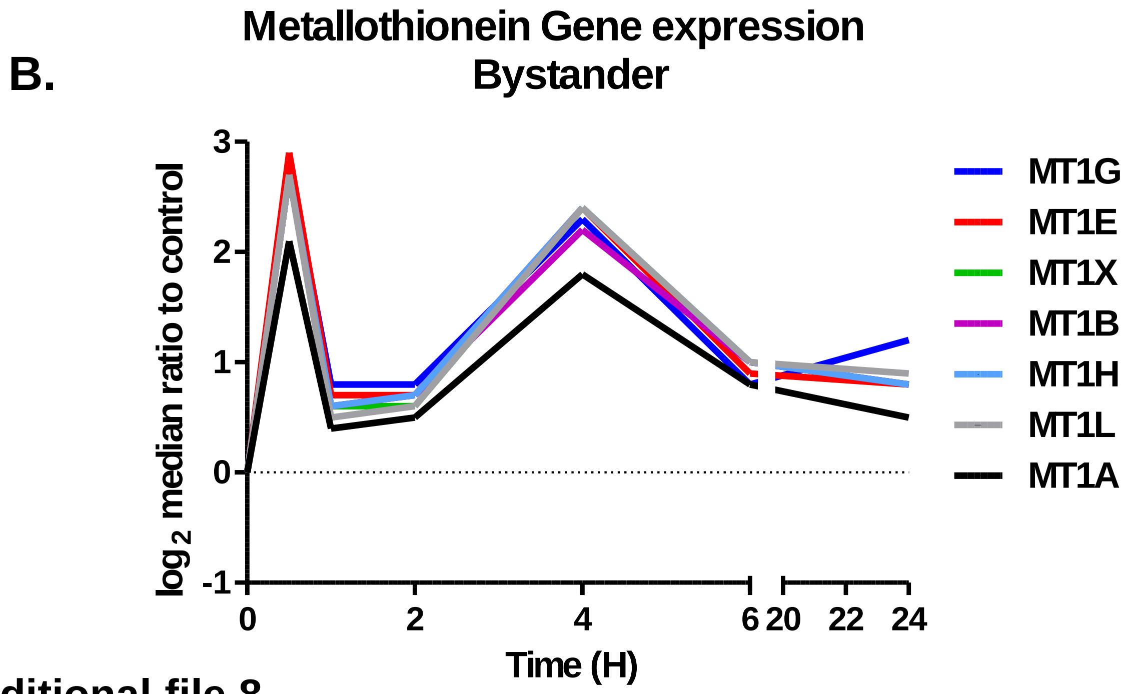

Additional file 8

A. Gene expression of Metallothionein genes after direct irradiation from FBPA Cluster 3 are displayed as time course plots of median $\log_2$ gene expression ratios in irradiated vs. non-irradiated controls. Data are medians across four independent biological replicates. Time is shown in hours (H).

B. Gene expression of Metallothionein genes after bystander treatment from FBPA Cluster 4 are displayed as time course plots of median $\log_2$ gene expression ratios in bystander vs. non-irradiated controls. Data are medians across four independent biological replicates. Time is shown in hours (H).
